# Correction: Maurer et al. Gut Microbial Disruption in Critically Ill Patients with COVID-19-Associated Pulmonary Aspergillosis. *J. Fungi* 2022, *8*, 1265

**DOI:** 10.3390/jof12040233

**Published:** 2026-03-24

**Authors:** H. Carlo Maurer, David Schult, Plamena Koyumdzhieva, Sandra Reitmeier, Moritz Middelhoff, Sebastian Rasch, Markus List, Klaus-Peter Janssen, Katja Steiger, Ulrike Protzer, Roland M. Schmid, Klaus Neuhaus, Dirk Haller, Michael Quante, Tobias Lahmer

**Affiliations:** 1 Department of Internal Medicine II, Klinikum Rechts der Isar, School of Medicine, Technical University of Munich, 81675 Munich, Germany; 2 ZIEL Institute for Food & Health, School of Life Sciences, Technical University of Munich, 85354 Freising, Germanyneuhaus@tum.de (K.N.);; 3 Chair of Nutrition and Immunology, School of Life Sciences, Technical University of Munich, 85354 Freising, Germany; 4 Chair of Experimental Bioinformatics, School of Life Sciences, Technical University of Munich, 85354 Freising, Germany; 5 Department of Surgery, Klinikum Rechts der Isar, School of Medicine, Technical University of Munich, 81675 Munich, Germany; 6 Institute of Pathology, School of Medicine, Technical University of Munich, 81675 Munich, Germany; 7 Institute of Virology, School of Medicine and Helmholtz Zentrum Munich, Technical University of Munich, 81675 Munich, Germany; 8 Department of Internal Medicine II, School of Medicine, University Hospital of Freiburg, 79106 Freiburg, Germany

Changes have been made to the original publication [1]. Supplementary Table S2 (per patient information from the MRI COVID-19/CAPA cohort) has been removed from the original publication and made available on reasonable request. This table contained de-identified or effectively anonymized patient information that added no value to the publication. The table has been deleted and can be requested from the corresponding author if required. This measure does not change the results or the thesis of the paper. Accordingly, the following formal (non-substantive) changes have been made to the manuscript:

(1) Results Section: “A basic summary of clinical characteristics and patient-level information is provided in Supplementary Tables S1 and S2” changed to “A basic summary of clinical characteristics is provided in Supplementary Table S1. Pseudonymized per-patient data to reproduce our findings are available on request”.

“Suppl. Table S3 and Suppl. Figure S1A” changed to “Suppl. Table S2 and Suppl. Figure S1A”.

“Suppl. Table S3 and Suppl. Figure S1B” changed to “Suppl. Table S2 and Suppl. Figure S1B”.

“Suppl. Table S3” changed to “Suppl. Table S2”.

“Suppl. Table S4” changed to “Suppl. Table S3”.

(2) Supplementary Materials Section: “Table S2: Per patient information from MRI COVID-19/CAPA cohort” was deleted.

“Table S3” changed to “Table S2”. “Table S4” changed to “Table S3”.

(3) Data Availability Statement: “De-identified clinical annotations for each of the 42 patients are provided as supplemental data tables” changed to “De-identified clinical annotations for each of the 42 patients from MRI COVID-19/CAPA cohort are provided on request”.

“Both data types will be made available without access restrictions upon publication” was deleted.

The authors state that the scientific conclusions are unaffected. This correction was approved by the Academic Editor. The original publication has also been updated.

## References

[1] Maurer H.C., Schult D., Koyumdzhieva P., Reitmeier S., Middelhoff M., Rasch S., List M., Janssen K.-P., Steiger K., Protzer U. (2022). Gut Microbial Disruption in Critically Ill Patients with COVID-19-Associated Pulmonary Aspergillosis. J. Fungi.

