# Supplementary material for: Gut Microbial Disruption in Critically Ill Patients with COVID-19-Associated Pulmonary Aspergillosis
Source: J Fungi (Basel). 2022 Nov 30;8(12):1265. doi: 10.3390/jof8121265 (PMC9787122; doi:10.3390/jof8121265)
Supplement: Supplementary file 1 [file jof-08-01265-s001.zip › Supplementary Figures.pdf]

Supplementary figures

Supplementary Figure 1

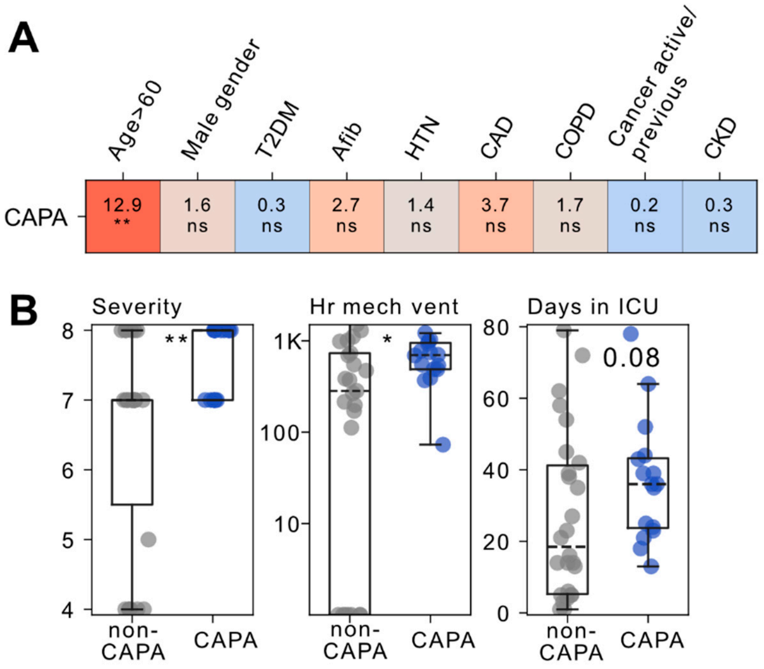

Supplementary Figure 1. (A) Association of COVID-19 associated pulmonary aspergillosis (y-axis) and the indicated clinical covariates (x-axis). For each combination and cell, Odds ratio (OR, upper number) and *p*-value code (from a two-sided Fisher's exact test) are depicted. Cells are colored by *p*-value and the sign of log(OR). (B) WHO disease severity, hours of mechanical ventilation and days treated in an ICU according to CAPA status. In boxplots, the box ranges from Q1 (the first quartile) to Q3 (the third quartile) of the distribution and the range represents the IQR (interquartile range). The median is indicated by a dashed line across the box. The “whiskers” on box plots extend from Q1 and Q3 to 1.5 the IQR. Unless otherwise specified, *p*-values are derived from two-tailed Mann-Whitney-U tests. *** $p <= 0.001$; ** $p <= 0.01$; * $p <= 0.05$; ns, not significant.

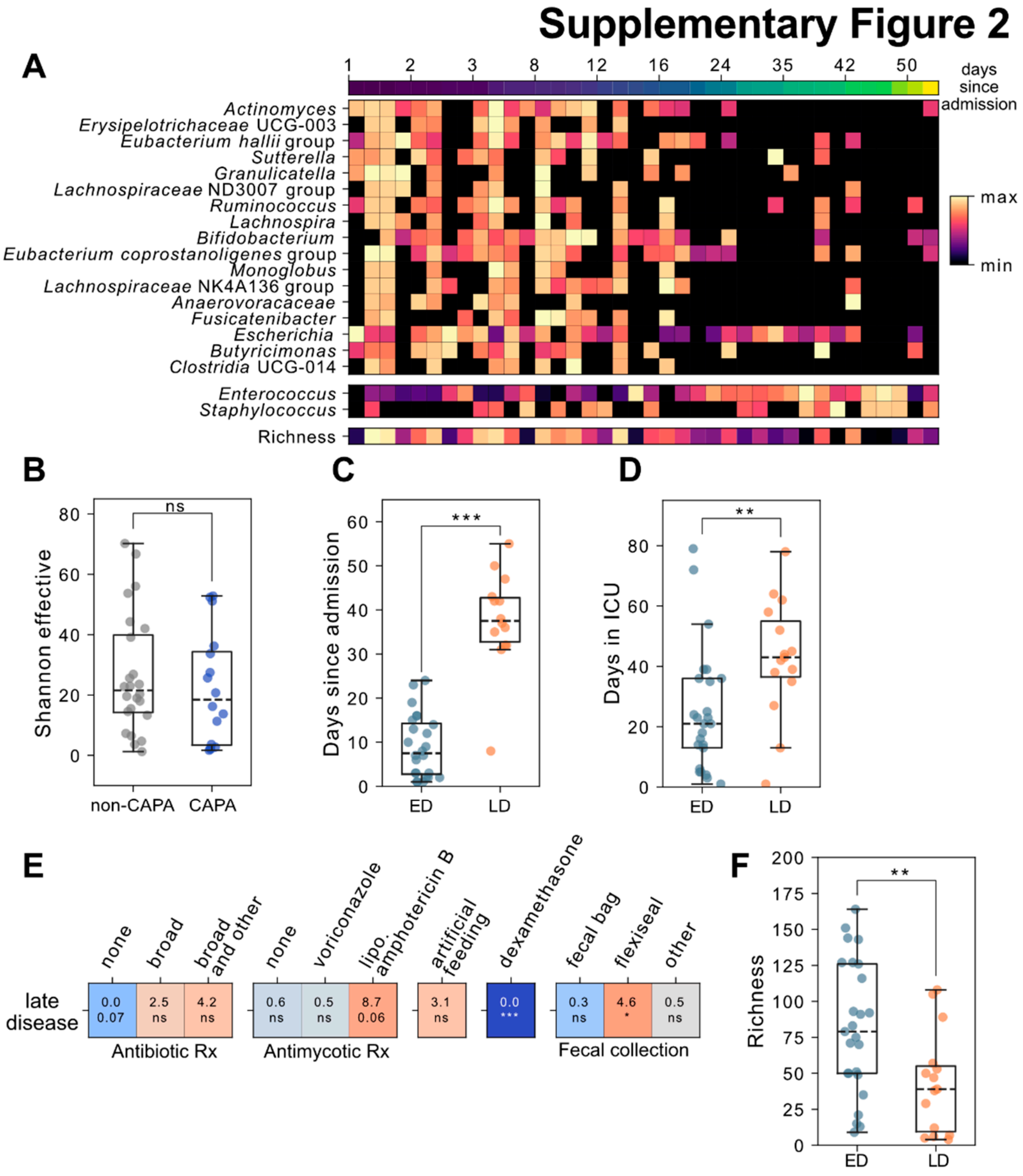

Supplementary Figure 2. (A) Heatmap representing quantile normalized abundance measures for the indicated genera and species richness (y-axis) ordered according to the time passed since hospital admission (x-axis). (**B**) Shannon effective number as a measure of alpha diversity among stool samples from COVID-19 patients with or without CAPA (**C**) Days since admission to the hospital according to COVID-19 disease stage (ED, early disease vs. LD, late disease) (**D**) Days spent in the ICU for patients with early and late COVID-19 disease (ED, early disease vs. LD, late disease) (**E**) Association of antibiotic and antimycotic treatments, feeding type and stool collection method (x-axis) and late COVID-19 disease. For each combination and cell, Odds ratio (OR, upper number) and *p*-value code (from a two-sided Fisher's exact test) are depicted. Cells are colored by *p*-value and the sign of log(OR). (F) Richness among microbial stool samples in early vs. late COVID-19 disease. In boxplots, the box ranges from Q1 (the first quartile) to Q3 (the third quartile) of the distribution and the range represents the IQR (interquartile range). The median is indicated by a dashed line across the box. The "whiskers" on box plots extend from Q1 and Q3 to 1.5 the IQR. Unless otherwise specified, *p*-values are derived from two-tailed Mann-Whitney-U tests. *** $p <= 0.001$; ** $p <= 0.01$; * $p <= 0.05$; ns, not significant.
